# A Multiple change-point detection framework on linguistic characteristics of real versus fake news articles

**DOI:** 10.1038/s41598-023-32952-3

**Published:** 2023-04-13

**Authors:** Nikolas Petrou, Chrysovalantis Christodoulou, Andreas Anastasiou, George Pallis, Marios D. Dikaiakos

**Affiliations:** 1 Computer Science Department, University of Cyprus, Nicosia, Cyprus; 2 Department of Mathematics and Statistics, University of Cyprus, Nicosia, Cyprus

**Keywords:** Computer science, Scientific data, Software, Statistics

## Abstract

Extracting information from textual data of news articles has been proven to be significant in developing efficient fake news detection systems. Pointedly, to fight disinformation, researchers concentrated on extracting information which focuses on exploiting linguistic characteristics that are common in fake news and can aid in detecting false content automatically. Even though these approaches were proven to have high performance, the research community proved that both the language as well as the word use in literature are evolving. Therefore, the objective of this paper is to explore the linguistic characteristics of fake news and real ones over time. To achieve this, we establish a large dataset containing linguistic characteristics of various articles over the years. In addition, we introduce a novel framework where the articles are classified in specified topics based on their content and the most informative linguistic features are extracted using dimensionality reduction methods. Eventually, the framework detects the changes of the extracted linguistic features on real and fake news articles over the time incorporating a novel change-point detection method. By employing our framework for the established dataset, we noticed that the linguistic characteristics which concern the article’s title seem to be significantly important in capturing important movements in the similarity level of “Fake” and “Real” articles.

## Introduction

Fake news are defined as stories that “describe events in the real world, typically by mimicking the conventions of traditional media reportage, yet known by their creators to be significantly false, and transmitted with the combined goals of being widely re-transmitted and of deceiving at least some of its audience”^1–3^. The term “fake news” is used in the literature to describe misinformation and disinformation that take place on the Internet and in online social media platforms like Facebook, Twitter, and Reddit, with “misinformation” referring to the spread of falsehood regardless of intent, and “disinformation” characterizing the “deliberate falsehood spread to deceive and cause harm”^4^. Several studies have demonstrated that state and non-state actors increasingly weaponize social media to spread fake news in the context of information-warfare operations of unprecedented scale and velocity, with a negative impact on societies and the democratic process^5–7^. The explosive growth and the impact of fake news on real-world events, such as the 2016 US presidential election and the COVID-19 pandemic, have raised the need for techniques and tools to identify fake news and mitigate their spread and penetration at scale and speed commensurate with their online spread.

To combat fake news, many research efforts^8^ are pursuing: (i) application of knowledge-based perspectives to identify falsehoods contained in online content; (ii) the detection of linguistic traits, writing style, format, and sentiment that are typical of fake content, and (iii) the detection of sources that disseminate fake news, such as web-sites and social media accounts. The identification of falsehoods is often supported by active citizens who identify suspicious posts, gather evidence to highlight false claims therein, and report them to fact-checking websites. Efforts to identify fake news in an automated manner analyze large datasets of both genuine and fake news articles to extract linguistic characteristics, select features that are useful for training fake-news detection models, train classification models (logistic regression, random forest, neural networks, etc.) with selected features, and apply models that provide the best results^9^. Careful feature engineering is required to select and extract appropriate textual or latent features in order to build and train effective and efficient models: elimination of ineffective features leads to lighter classification models that require less computational resources for training and deployment, as well as more accurate results. Trained models are then deployed on social media platforms or on end-user devices (browsers, applications) and used to classify articles as fake or not, and flag or filter-out falsehood. The predictive power of models can be improved further by taking into account additional features, which represent the profile and “reputation” of news-media sources and distributors, such as websites and social media accounts^10,11^.

In summary, a key challenge in the automated detection of fake news is the identification of linguistic features that help differentiate between fake and authentic news articles. Prior work has shown that the text of fake news is characterized by informal, sensational, affective language since their ultimate goal is to attract attention for short-term financial or political gains rather than to build a long-term relationship with readers^12,13^. Also, that significant deviations exist between linguistic profiles of authentic versus fake news articles^11,14^.

However, since human languages and the use of words evolve steadily with time^15^, it is expected that linguistic features of fake news will also change with time. Our goal is to explore this hypothesis and investigate possible differences in the temporal evolution of linguistic features of fake versus authentic articles. To this end, we develop a framework that (i) extracts the most informative linguistic features of news articles; (ii) classifies articles to various categories based on their content; (iii) applies a novel change-point detection method to detect the temporal evolution of linguistic features extracted, and (iv) compares differences between the language evolution of real and fake news. Change-point detection is an active area in statistical research, which features algorithms that segment data into smaller, homogeneous parts using flexible statistical models that can adapt to non-stationary environments. Due to the natural heterogeneity of data in many real-life problems, novel change-point detection methodologies have been applied in a wide range of application areas, from credit scoring^16^ and cyber security^17^ to finance^18^.

In this paper we investigate the following research questions: (i) Which linguistic characteristics mainly appear informative in identifying a fake news article? (ii) Taking into account that language is evolving, how do these characteristics change over time? (iii) Can we detect the occurrence of such changes? To address these research questions:

- We introduce DECLARE, a novel framework that retrieves articles from trusted and suspicious domains (where the majority of them are in UK and US) and conducts an extensive linguistic analysis to investigate (i) which are the most informative linguistic features for detecting fake news articles, and (ii) how the linguistic features of fake news articles change over the years.
- We develop and release a new publicly available large dataset from news articles, which have been published between 2009 and 2019 (more details can be found in the "Data availability" section). This dataset consists of 534 linguistic features of time-stamped fake and real news-articles. The feature selection process uses a lasso regression model and is described in more detail in the "Framework" section.

The rest of this article is organized as follows: The "Related work" section reviews previous works on exploiting linguistic characteristics that are common in fake news, the "Framework" section describes the proposed framework that has been used for this analysis. The results are presented and discussed in the "Results" and "Conclusion" sections.

## Related work

In this section, we summarize works focusing on “style-based fake news”, namely news articles with quantifiable features that represent linguistic traits, writing style, format and sentiment, and which can help distinguish fake news content from authentic news. A comprehensive survey of such approaches is given in^8^. These methods focus on extracting features that capture language use^19^, sentiment and writing style^20^ or combinations thereof^11^. Linguistic features are classified into three categories: (1) Stylistic features, which represent the syntax and textual style of articles’ headlines and content, with textual style reflected in features like the frequency of stop-words, punctuation, quotes, negations and words that appear in all capital letters, and syntax style reflected in the frequency of Part-of-Speech (POS) tags in the text, and the use of proper nouns and capital words in the titles^14^. (2) Complexity features, which aim at capturing the overall intricacy of articles and headlines. These features can be computed based on several word-level metrics that include readability indexes and vocabulary richness, and which achieve higher classification accuracy in contrast to other feature combinations^21,22^. (3) Psychological features, which are based on frequencies of words found in dictionaries associated to sentiment and psychological processes. Such words are found in dictionaries like LIWC, which are curated by human domain experts. The sentiment score is computed via the AFINN sentiment lexicon^23^, a list of English terms manually rated for valence^11^. The premise behind using psychological features is that fake content has been shown to be considerably more negative than authentic news^14,22^.

Other promising approaches include multi-class logistic regression for conducting stance classification^24^, graph-kernel based hybrid support vector machine classification of high-order propagation patterns in addition to semantic features such as topics and sentiments^25^, the combination of time-series-based features extracted from the evolution of news along with characteristics of the user accounts involved in the spreading of news^26^, and deep learning (DL)^27^. Finally, one of the most important recent advances in NLP, the DL-based BERT language representation model^28^, has also been used for fake news detection^3^.

To sum up, although there have been several approaches demonstrating the importance of writing-style features for the detection of fake news, there are hardly any studies exploring how the linguistic characteristics change over the years as well as to apply statistical change-point detection on fake news trends. Such a study will contribute towards understanding the fake news articles as well as improving the classification models for fake news detection.

## DECLARE framework

**Figure 1:**
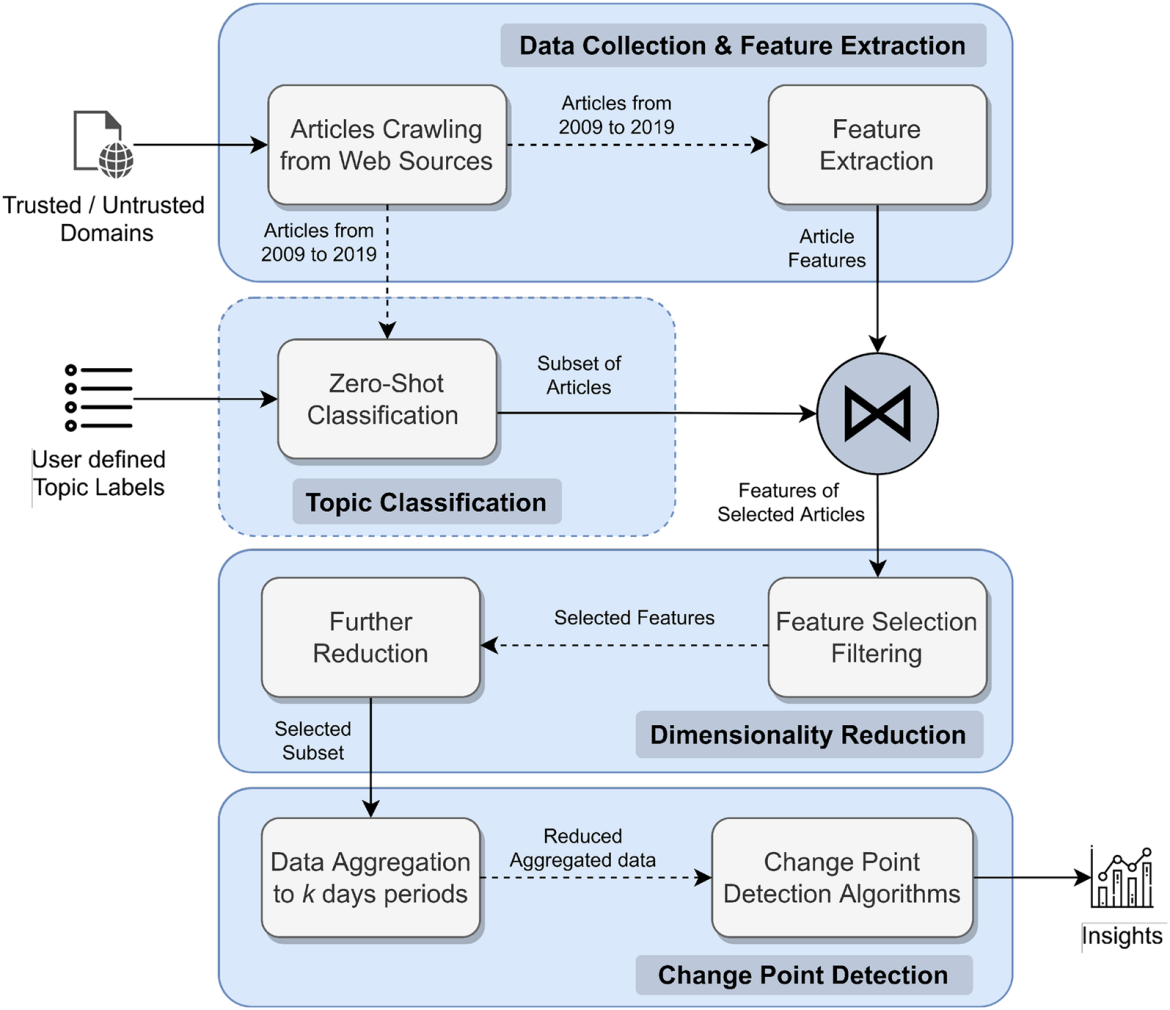
DECLARE: overview scheme of framework.

The proposed framework is partitioned into four different modules, as shown in Fig. 1. Initially, the articles are collected based on given URL domains (trusted/untrusted) and the feature extraction is accomplished. Next, the optional step of Topic Classification is performed in order to select articles of desired topics. Subsequently, Dimensionality Reduction is carried out for the extracted features of the selected articles, and finally multivariate, offline change-point detection is conducted on the data subset. For the rest of this section, the problem overview is formulated and the different segments of the work are examined in detail.

### Problem overview

We have a set of $K \in {\mathbb {N}}$ articles, which we denote by $A := \left\{ \varvec{a_1}, \varvec{a_2}, \ldots , \varvec{a_{K}} \right\}$. Each article $\varvec{a_i}$, $i = 1,2,\ldots , K$ is a vector of dimensionality $d+1$, with $d \in {\mathbb {N}}$. The first *d* elements of $\varvec{a_i}$ are the relevant values for each one of the *d* linguistic features employed. In addition, each news article $\varvec{a_i}$, $i = 1,2,\ldots , K$ is categorized as “Real” or “Fake”. Hence, the last element of each vector $\varvec{a_i}$ is a value in the set $\left\{ 0,1 \right\}$ and denotes the respective article’s class label, where the values of 0 and 1 correspond to the classes “Real” and “Fake”, respectively.

The problem that we deal with in this work is to study the attributes which most accurately characterize political articles, and to identify how they evolve over time. To do so, a set *B* which corresponds to those informative linguistic features is required. Therefore, after deciding these features, each article $\varvec{a_i}$, $i = 1,2,\ldots , K$ will become a vector of a reduced dimensionality $|B| \le d + 1$. Next, the articles are stamped by the time component $t \in \{1,...,T\}$, and thus multivariate data sequences will be constructed by utilizing the article set *A*; for more information regarding the construction of data sequences, refer to the "Change-point detection" section. Then, a multivariate change-point detection technique is employed to detect abrupt changes in the linguistic behavior of articles categorized as “Fake” compared to the behavior of those categorized as “Real”. Distinctively, the process shall reveal the estimated number of change-points, denoted by ${\hat{N}}$, while the estimated locations, sorted in an increasing order, are denoted by ${\hat{r}}_1, {\hat{r}}_2, \ldots , {\hat{r}}_{{\hat{N}}}$. In addition, for every estimated change-point location ${\hat{r}}_i$, $i = 1,2,\ldots , {\hat{N}}$, post-processing will be performed in order to determine the linguistic feature set $B_i$, with $B_i \subseteq B$, that contains the linguistic features in which the relevant change-point, ${\hat{r}}_i$, was discernible the most.

### Data collection and feature extraction

Regarding the Data Collection process, a list of collected articles was constructed by crawling the WebArchive^29^ for news articles published from 2009 through 2019. The articles were divided into untrusted and trusted ones, based on multiple sources. Initially, we used two pairs of domain credibility lists, which were originally formed in^30^. The first, contains domain names that usually publish Fake News and are highly scrutinized by fact-checking organizations, including Snopes, PolitiFact, and others. The latter includes high reputation domains, which have rarely or never been criticized by fact-checking sites. The lists contain both popular and uncommon domains with English content from newspapers as well from blogs. Since the use of only one source could have brought bias to the analysis, labels provided by the independent online fact-checking outlet MediaBiasFactCheck^31^ (MBFC) were also employed to verify the validity of these lists. MBFC specifies how often a domain publishes factual news by employing seven labels ranging from VERY LOW to VERY HIGH. We adopted the approach of Chen and Freire^32^, and we define “untrusted” domains as those with labels VERY LOW, LOW, MIXED, and “trusted” domains as those with VERY HIGH, HIGH, and MOSTLY FACTUAL. Thus, utilizing the knowledge of these lists and MBFC, the possibility of false positives was limited by discarding domains with discrepancies on their labels and domains which were not classified by MBFC. The procedure has resulted to 2152 untrusted and 256 trusted domains.

Eventually, the crawling procedure is the most vital and resource-demanding part of the data collection process. This component is responsible for crawling the domains inside the WebArchive efficiently. WebArchive has saved more than 583 billion web pages over time and provides the WayBackMachine service, which allows the traversal of a website through time. To crawhigh-reputatione, we used Scrapy^33^, a Python library, which is considered one of the fastest web-crawling tools, especially for complex crawling and scrapping applications.

The next step of the data collection process is to extract the actual article from the website. However, extracting information from various articles with different formats which are coming from different domains is a challenging procedure. To address this issue, the Newspaper3K^34^ library of Python was utilized. Specifically, Newspaper3K helps in building an article’s object including fields like the article’s title, body, and publish date in a way that does not depend on the orientation of the website. After the execution of the data collection pipeline, we perform data wrangling to remove duplicate or unreadable articles, as well as to fix formatting errors and remove any HTML code embedded in the text.

Finally, in order to analyse how text-linguistic characteristics differ between real and fake sources, we extract a plethora of different linguistic features to gather all the available knowledge from the text. To this end, we rely on a previous study^11^, which identified 534 features that capture Stylistic, Complexity and Psychological aspects of articles. Table 1 unveils a list summarizaing a subset of the employed features. This processing results in a large dataset, named LinCFNA, which consists of a total 320,960 time-stamped articles, both fake and real, each characterized by 534 different linguistic features.

### Topic classification

The initial corpus did not contain any labels regarding the topics of articles. To be able to select articles for further analysis that belong to particular topics (e.g., politics) we perform an automated topic classification to map all corpus articles to predefined topics of interest. To this end, we adopt Zero-Shot text classification^35^, a highly accurate method that allows classification to classes not used or seen during the model’s training^35,36^. Zero-shot classifiers leverage pre-trained language models, and therefore it can be thought of as an instance of transfer learning, which generally refers to using a model trained for one task in a different application than what it was originally trained for^37^. This is particularly useful for situations where there are little to no available labelled data. The original proposed textual entailment Zero-Shot method uses Natural Language Inference (NLI) based models as ready-made Zero-Shot text classifiers. In particular, the method converts the original text data into entailment data where, it considers a text sequence under classification as the NLI premise and constructs a hypothesis from each candidate label. For instance, in order to evaluate whether a sequence’s topic corresponds to the user-defined “politics” class, the model can extract the corresponding label probabilities. Regarding the task formulation and training procedure of the Zero-Shot textual models, as well examples of created hypotheses for modeling different aspects, details can be found in^35^. This approach was proven to be remarkably effective in many text classification tasks, particularly when used with large pre-trained models like BART^38^ or RoBERTa^39^. In this work, for the deployment of the NLI model, we employ the publicly available Hugging Face Transformers library^40^ along with the pre-trained BART-large model, developed by Meta (formerly Facebook)^41^. The specific model, which works without requiring any data other than the provided text, is fine-tuned for the Multi Genre Natural Language Inference corpus (MNLI) dataset, that contains ten different text categories^42^. The Hugging Face’s model pipeline employs both the pre-trained model as well as the pre-processing procedures that were performed during the fitting stage. Therefore, little to none text preprocessing is required during inference time. In addition, lemmatization or stemming are not required either, due to the fact these models are mostly trained on raw text, using WordPiece tokenizers^28^. To that extent, only a minimal text cleaning was performed prior to using the aforementioned pipeline by removing HTML code, fixing unicode errors and finally using special tokens for any embedded URLs or phone numbers.

In addition, LinCFNA contains observations of articles which could be considered to belong to more than one category. Therefore, we follow a multi-label approach. Specifically, unlike normal classification tasks where class labels are mutually exclusive, multi-label classification involves predicting multiple mutually non-exclusive labels, where the labels are considered independent. The probabilities are normalized for each candidate label by applying a softmax of the “Yes” versus the “No” scores. That way, for a given article, the model produces a vector of independent probability values for each candidate label. An article is said to belong to a predefined candidate label as long as the respective probability is larger than 0.5.

In this work, our analysis focused on political news articles, since we would like to study fake news around political articles. Thus, each article $\varvec{a_i}$, $i = 1,2,\ldots , K$ is classified as part of the “Politics” class and kept for further analysis as long as the probability of $\varvec{a_i}$ being a political article is greater or equal than *Q*, with $Q = 0.75$. We used $Q = 0.75$ instead of the model’s default threshold 0.5 for two reasons. Firstly, since the accuracy of the Zero-Shot approach could not be directly observed, and therefore bias or ambiguous labels could have been created during the analysis phase due to its non-supervised nature, we used a larger threshold than the default one in order to discard the articles with the most controversial labels. Secondly, by using a very large value of *Q*, for example, $Q = 0.9$, led to significantly fewer articles classified as political, which then created problems in the change-point detection part that requires the existence of observations for every time point within the period tested. Therefore, *Q* was chosen to keep in mind that a plethora of observations were selected, while the most ambiguous observations were not kept for further analysis, in order to produce more representative and robust results. Out of the original 320,960 observations of LinCFNA, the aforementioned procedure led to a set *A* of 87, 066 articles about politics. Even though the non-political or ambiguously selected articles were limited by adjusting the value of *Q*, we further examined the validity of the selected articles for a random sample of them. Specifically, since the ground-truth labels of the “Politics” class were not present, to examine the validity of the political labels assigned, we sampled and checked the correctness of 100 out of the total assigned political articles. Based on our evaluation, around $90\%$ of the articles were correctly assigned the “Politics” class label. Based on the set *A*, we construct the matrix $X \in {\mathbb {R}}^{|A| \times 535}$ which comprises the linguistic representation of the selected political articles along with their validity labels.

### Dimensionality reduction

The aim of dimensionality reduction is to reduce the size of the $X \in {\mathbb {R}}^{|A| \times 535}$ matrix by pruning some of the initial 534 features, and end-up with a matrix $X_R \in {\mathbb {R}}^{|A| \times (d+1)}$ of a lower dimensionality, which contains only $d \in {\mathbb {Z}}^{+}$ informative and supportive features that reveal fruitful information about the credibility of topic specific articles. A matrix with a lower dimensionality will improve substantially the effectiveness of the upcoming change-point detection analysis. As Fig. 1 illustrates, feature reduction procedure consists of two different phases. The first applies filtering whereas the second applies embedded feature selection and component based reduction techniques.

During the first phase, we remove 29 features with very low sample variance, namely attributes that have the same value in nearly all observations, which lead to a new reduced feature matrix $X_F \in {\mathbb {R}}^{|A| \times 505}$. The removed attributes of LinCFNA along with their sample variance are included in Table S1 in the Supplementary Tables Section of the online supplement. During the second phase, instead of just using a filtering evaluation function that relies solely on properties of the features (e.g. the variance threshold or Pearson’s correlation coefficient), we apply two individual non-filtering methods. In the first one, with $X_{[j]}$ denoting the jth column of the matrix *X*, the articles’ labels in $X_{[506]}$ were also employed in a supervised embedded method for a further selection of important features, while the second approach relied on a component based reduction technique.

For the first approach, we employed a classification model that utilizes a feature selection mechanism during the algorithm’s modelling execution. Such mechanisms generally aim at the inclusion of predictors that strongly aid the generalization of unseen data, by preventing the learning algorithm from overfitting the training dataset. Embedded methods perform feature selection in the process of training and are usually specific to given learning machines^43^. Typical embedded methods include various types of tree based algorithms, like CART, C4.5, and Random Forest trees^44^, but also other algorithms such as logistic regression variants^45^. Among the embedded methods, there are regularized models which perform feature weighting based on objective functions that minimize fitting errors while forcing the predictor coefficients to be exactly zero. These methods, which are based on the penalization of the $L_1$ norm (Lasso), induce penalties to features that do not contribute to the model and they usually work with linear classifiers, such as Support Vector Machines or Logistic Regression^46^.

Even though many embedded feature selection options are available, for this specific work, we adopt a logistic regression model penalized using the $L_1$ norm, to obtain a robust classifier with sparsity in the coefficients^47^. Additionally, regarding the strength of regularization, we compute the regularization path at a grid of values in a stratified *k*-fold Cross Validation (CV) manner for the regularization parameter $\lambda$, which controls the overall strength of the lasso penalty. The choice of the number of folds, *k*, is usually 5 or 10, but there is no formal rule^48^. In our work, different values for the number of folds have been employed since there is a trade-off on the choice of *k*; larger values lead to much higher computational complexity and lower prediction errors, while smaller values lead to more computationally efficient results with possibly higher prediction errors. Therefore, we took $k \in \left\{ 5, 10, 25\right\}$, and since the results were extremely similar in terms of the selected features, we carried out the analysis with $k = 10$. With respect to $\lambda$, since larger values of this hyperparameter produce solutions with more sparsity in the coefficients, for assessing the different amount of features, the classifier was evaluated on the left-out folds with different values of $\lambda$. The procedure exploited the $F_1$ score evaluation metric, since it combines both the precision and recall of a classifier into a single metric by taking their harmonic mean^49^. We selected a model size with a penalty large enough, which though still allows to accurately classify observations, in respect to the $F_1$ score metric. The obtained $F_1$ scores for the different regularization parameter values, $\lambda$, can be found in Figure S1 in the Supplementary Figures Section of the online supplement. Based on the aforementioned figure and the slope of the obtained curve, the regularization parameter value to be used was chosen as the point on the x-axis where the absolute value of the slope in the curve is at its minimum; not long after this point, we see that there is a steep drop on the $F_1$-scores. Finally, this approach led to a new reduced feature matrix $X_{Lasso}$ that consists of 34 unique features, which are summarized in Table 1.

We selected the aforementioned lasso approach because it can exploit the sparsity in the input matrix $X_F$, and since, for high-dimensional data, it is extremely fast over other feature selection methods. Therefore, it was computationally convenient to employ the lasso approach with different settings in order to explore how different model sizes and informative features could perform in predicting the labels of the articles. Apart from the lasso method, which is easily interpretable, we also utilize a component-based approach. The intuition behind the use of another dimensionality reduction method was to study the robustness of the framework and compare the different estimated change-point locations that would have been produced by the two methods.

As a component-based approach, we employed Principal Component Analysis (PCA), mainly because it was perceived that there are variables which are strongly correlated; therefore, feature reduction has been essential, without losing critical information. PCA is an unsupervised dimensionality reduction technique, which ignores the class labels of the articles. Instead, PCA focuses on capturing the direction of maximum variation in the data set, by defining an orthogonal projection of the data onto a lower dimensional linear space, known as the principal subspace, such that the variance of the projected data is maximized^50^. Regarding the selected number of components, given that the upcoming multivariate Change-point Detection module first performs a data aggregation operation that accumulates the data points to *k* day periods, considerably reducing the number of observations to 255, the number of components needed to be an order of magnitude less than the length of the data sequence; otherwise, we would potentially run into high-dimensionality and large computational complexity issues. We worked on taking the first 10, 20, and 30 principal components with the results being extremely similar due to the robustness of the proposed change-point detection algorithm; for more information on this, see Section Results of^51^. Therefore, in the article, we present the obtained results for the most parsimonious case, meaning when the first 10 principal components were selected. Ultimately, this approach led to a new reduced feature matrix $X_{PCA}$ that consists of 10 features, which are linear combinations of the original ones. The results acquired when we used the first 20 or 30 principal components are given in the Supplementary Methods Section of the online supplement.

### Change-point detection

**Figure 2:**
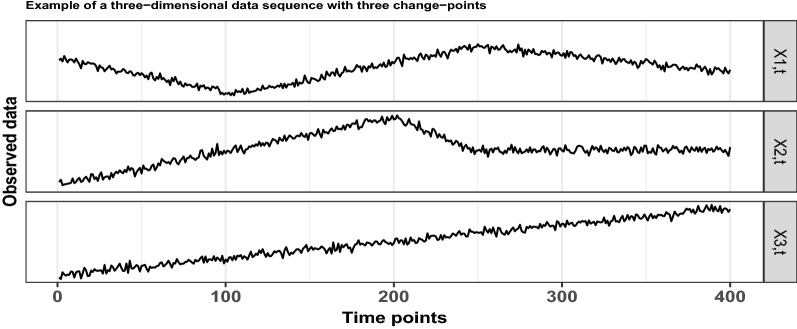
An example of a three dimensional data sequence with piecewise-linear structure, that undergoes three changes in its first derivative at locations $r_1 = 106, r_2 = 200$ and $r_3 = 248$.

Based on whether we have full knowledge of the data to be analysed, change-point detection is split into two main categories; offline detection, where the data are already obtained, and online detection, in which the observations arrive sequentially at present. With respect to the dimensionality of the data, change-point detection can be further separated into algorithms that act only on univariate data and to those that are suitable for change-point detection in multivariate, possibly high-dimensional data sequences. In this section, the focus is on offline change-point detection and with $p \in {\mathbb {N}}$ denoting the dimensionality of the given data, the model in full generality is given by

1 $$\begin{aligned} \varvec{X_{t}} = \varvec{f_{t}} + \Sigma \varvec{\varepsilon _{t}}, \quad t=1, \ldots , T, \end{aligned}$$

where, at each time point *t*, $\varvec{X_{t}} \in {\mathbb {R}}^{p\times 1}$ are the observed data and $\varvec{f_{t}} \in {\mathbb {R}}^{p \times 1}$ is the underlying, unknown, *p*-dimensional deterministic signal which undergoes structural changes at certain, unknown points. The matrix $\Sigma \in {\mathbb {R}}^{p \times p}$ is diagonal, while the noise terms $\varvec{\varepsilon _{t}} \in {\mathbb {R}}^{p \times 1}$ are random vectors with mean the zero vector and covariance the identity matrix. In the current manuscript, we are looking for changes in the vector of first order derivatives, or, in other words, changes in the slope of any of the univariate component data sequences. In Fig. 2, we graphically provide an example of a three-dimensional data sequence of length $T = 400$. The diagonal elements of the matrix $\Sigma$ are all equal to 7, while $\varepsilon _{t,i}$ follow the standard Gaussian distribution for $i=1,2,3$. There are three change-points in the slope at locations $r_1=106, r_2=200$ and $r_3=248$. To be more precise, the first two component data sequences have two change-points each; for $X_{1,t}$ at locations $t=106$ and $t=248$, while for $X_{2,t}$ at locations $t = 200$ and $t = 248$. There are no change-points in $X_{3,t}$.

A recently developed change-point detection method, called Multivariate Isolate-Detect (MID), is applied to the given multivariate data sequence $\varvec{X_{t}}$; the component data sequences are related to the extracted features; more details are given in Steps 1–3 below. MID has been introduced in^51^ and is a generic technique for the detection of the number and the location of multiple structural changes in the behaviour of given multivariate, possibly high-dimensional data. It provides maximal detection power by testing for change-points into intervals that contain at most one change-point; this specific isolation technique was first introduced in the Isolate-Detect (ID) methodology of^52^. The main idea is that for the observed data sequences $x_{t,j} t=1,\ldots , T,\quad j=1,\ldots ,p,$ and with $\lambda _{T}$ a positive constant, MID first creates two ordered sets of $K=\lceil T/\lambda _{T}\rceil$ right- and left-expanding intervals. For $i =1,\ldots , K$, the ith right expanding interval is $R_i=[1,\min \left\{ i\lambda _T, T\right\} ]$ while the ith left-expanding interval is $L_{i}=[\max \left\{ 1, T-i\lambda _{T}+1\right\} ,T].$ We collect these intervals in the ordered set $S_{RL}=\{R_1,L_1,R_2,L_2,\ldots ,R_K,L_K\}$. The algorithm first acts on the interval $R_1 = [1, \lambda _T]$ by calculating, for every univariate component data sequence, an appropriate contrast function value for the $Q \in {\mathbb {Z}}^{+}$ possible change-point candidates in this interval (details on suitable contrast functions to be used are given in Section"Data collection and feature extraction"of^52^). This process will return *Q* vectors $\varvec{y_j}, j=1,\ldots ,Q$ of length *p* each; for example, the elements of $\varvec{y_1} \in {\mathbb {R}}^{p}$ will be the contrast function values related to the first change-point candidate in $R_1$, for each of the *p* component data sequences, the elements of $\varvec{y_2} \in {\mathbb {R}}^{p}$ will be the relevant values for the second candidate in $R_1$, and so on. The next step is to apply to each $\varvec{y_j}$ the mean-dominant norm $L: {\mathbb {R}}^p \rightarrow {\mathbb {R}}$ with $L(\varvec{y_j}) = \frac{1}{\sqrt{p}}\sqrt{\sum _{i=1}^p y_{j,i}^2}$ where $y_{j,i} \ge 0, \;\forall i, j$. Applying $L(\cdot )$ to each $\varvec{y_j}$, will return a vector ${\varvec{v}}$ of length *Q*. We identify ${\tilde{b}}_{R_1}$ := $\textrm{argmax}_j\left\{ v_j \right\}$. If $v_{{\tilde{b}}_{R_1}}$ exceeds a certain threshold, which has been explicitly derived in^51^, then ${\tilde{b}}_{R_1}$ is taken as a change-point. If not, then the process tests the next interval in $S_{RL}$. Upon detection, the algorithm makes a new start from the end-points of the expanding interval where the detection occurred.

Under Gaussianity of the noise terms $\varvec{\varepsilon _t}$, MID has been proven to be consistent in accurately estimating the true number and locations of the change-points; for further details, please see Section "Related work" of^51^. However, in practice, both ID and MID (which is employed in the current multivariate framework) have been shown to be robust and to exhibit very strong performance in scenarios where we could have either auto-correlated or heavy-tailed noise; for more details on such robustness results, please see Sections "Results" and "Conclusion" of^52^.

For the purposes of this paper, the matrices $X_{Lasso}$ and $X_{PCA}$, which are derived from the dimensionality reduction stage explained in the previous section, will be used in creating $\left\{ {\varvec{X}}_t \right\} _{t=1,2,\ldots ,T}$ of Eq. (1), to which the algorithm MID will be applied. The three main steps that have been followed are given below.

*Step 1* From the given matrix ($X_{Lasso}$ or $X_{PCA}$, depending on the dimensionality reduction technique employed), two smaller matrices are first created splitting the data into those obtained from articles categorised as “Fake” and those obtained from articles categorised as “Real”. From now on, the notation employed for the aforementioned two matrices is $Y_F$ and $Y_R$, respectively.

*Step 2* To have more information, and therefore higher detection power, we worked on biweekly data. Aggregation over time is essential in order to avoid direct utilization of raw, misleading observations. In addition, it was important to achieve aggregation of the observations in a way that both fake and real data would appear within every period. Regarding the selected aggregation interval, by experimenting we confirmed that aggregating into two-week periods (specifically 14-day periods) allowed us to have sufficient information for both fake and real news within every time period used for the change-point detection part of our proposed framework. Therefore, the values for the various features in the matrices $Y_F$ and $Y_R$ have been aggregated over a period of 14 days. For the aggregation step, we employed the sample average of the relevant values for each feature. To be more precise, with $p \in {\mathbb {N}}$ being the number of features used, since there are 255 periods of 14 days in our data, after this second step, two matrices, $Y_R^*$ and $Y_F^*$ of dimensionality $255 \times p$ are created, which contain aggregated information for articles classified as “Real” or “Fake”, respectively.

*Step 3* The matrix $Y_{diff} = Y_R^* - Y_F^* \in {\mathbb {R}}^{255 \times p}$ is created.

After the above three steps have been completed, we will be under the scenario of looking for abrupt changes in the trend of ${\varvec{X}}_t$ as in (1), where specifically now ${\varvec{X}}_t = [Y_{diff}]_{[t]}, t=1,2,\ldots ,255$, where $[Y_{diff}]_{[t]} \in {\mathbb {R}}^{p \times 1}$ is the $t^{th}$ row of the matrix $Y_{diff}$. We work on the differences between the values of the characteristics for “Fake” and “Real” articles in order to capture significant deviations in the comparative behaviour between articles from the two aforementioned categories. This will provide an indication of attempts from unreliable news agencies to write articles in such a way that resembles those published from trusted news sites.

### Ethical approval

The authors declare that no human participants were involved in the study or data acquisition.

## Results

### Informative Linguistic characteristics of political articles

Applying the lasso approach in the DECLARE framework as described above, we extract the most informative linguistic features which aid in identifying whether a political article is fake or not. Table 1 unveils the list of the 34 important features which were most informative in linearly separating the articles to fake and real. The results support that most of the linearly informative characteristics are related to psychology. That indicates the contrast between fake and real political articles, in psychological factors that demonstrate the intention of the writers to convince readers that the content is realistic by using certain mechanisms (e.g. by emotionally influencing readers or by overdramatizating certain events through Persuasive Language)^53,54^. The extracted feaure AFINN sentiment score^23^ was indeed expected to be informative, since sentiment analysis on fake news articles had revealed that fake news tends to contain increased negative emotional language^14,22^. Moreover, the many selected features from the widely used Linguistic Inquiry and Word Count (LIWC) dictionary, demonstrate that fake and real news differentiate in terms of choosing the words that reveal psychometrics characteristics.

Furthermore, the results reveal that most of the significant stylistic attributes are structural features and are linked to the title of an article. Interestingly the knowledge which can be extracted from uppercase text is certainly fruitful as it was found that three of the important features are based on uppercase letters. This observation is linked with previous studies around fake news, as fake news were found to be more dramatic with copious use of uppercase letters to make it a click-bait for the readers^55^. Finally, regarding characteristics which expound the complexity and writing style of articles, we spotted that a group of word-level readability and vocabulary richness measures are helpful in distinguishing fake and non-fake political articles, which concur with identical findings in^21,22^.

### Evolution of linguistic characteristics

By taking into account the established results of the lasso-based model, we showed that certain characteristics differ between the fake and non-fake political articles. However, since the time-component is involved and due to the fact that both language and writing are continuously evolving, these differences between the informative characteristics are expected to be changing as well. Applying the MID change-point detection algorithm with its default parameter values, the following results were obtained:

For the lasso-based dimensionality reduction method, four change-points were detected. Those correspond to the following 14-day periods; in parentheses we provide the features in which these changes were apparent the most:

- *First change-point* Period: 2011-03-02 until 2011-03-15 (“Title_total_number_of_sentences”, “Title_ratio_uppercase”).
- *Second change-point* Period: 2011-10-26 until 2011-11-08 (“LIWC_Negate”).
- *Third change-point: Period* 2014-02-12 until 2014-02-25 (“Title_total_number_of_sentences”).
- *Fourth change-point* Period: 2016-09-07 until 2016-09-20 (“Title_total_number_of_sentences”, “Title_ratio_uppercase”, “Title_avg_number_of_all_caps_per_sentence”, “RID_Secondary_Social_Behavior”, “RID_Secondary_Temporal_Repere”, “LM_Weak_Modal”, “Sichel’s Vocabulary Richness”).

**Figure 3:**
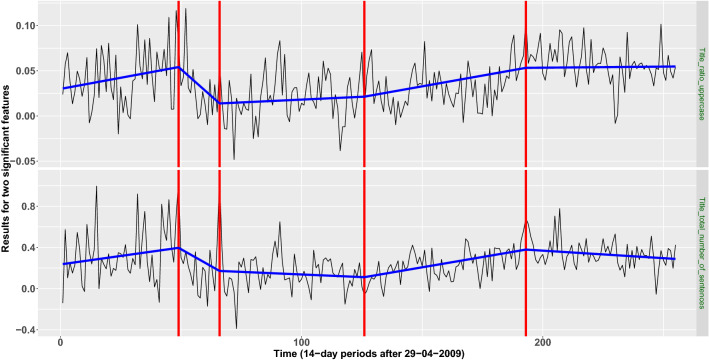
The data sequences for “Title_total_number_of_sentences” (bottom row) and “Title_ratio_uppercase” (top row), the estimated change-point locations (red vertical lines), and the fitted continuous and piecewise-linear signals (presented with blue colored lines) obtained by the MID change-point detection algorithm.

We observe that two features, “Title_total_number_of_sentences” and “Title_ratio_uppercase”, seem to be more important than the rest in capturing important movements in the similarity level of “Fake” and “Real” articles. Figure 3 presents the results for the aforementioned two features.

In an attempt to provide a possible explanation of the estimated change-point locations, looking at the movements of the estimated signals in Fig. 3 between consecutive segments, we observe that there is a significant drop in the value of their slopes after the first and the last change-point. Changes in the slope of such nature indicate that after the aforementioned two change-points, the distinction between fake and real articles became less apparent, meaning that there had possibly been an intentional, successful attempt from news agencies that produce fake articles to resemble more the writing style of trusted and long-established agencies.

**Table 1:** Informative linguistic features extracted from lasso logistic-regression.

Feature	Definition
**Complexity features**	
Automated readability index	$0.39\left( \frac{\text {Total} \# \text { of Words}}{ \text{ Total } \#\text { of Sentences }}\right) +11.8\left( \frac{ \text{ Total } \# \text {of syllables }}{ \text{ Total } \#\text { of words }}\right) -15.59$
Coleman-Liau readability index	$5.88 \left( \frac{\text{Total } \#\text { of Letters}}{\text{ Total } \#\text {of Words}}\right) -29.6* \left( \frac{\text{ Total } \#\text {of Sentences}}{\text{ Total } \#\text {of Words}}\right) - 15.8$
Sichel’s Vocabulary Richness	Total # of Happaxdilsegomena /Total #of Words
**Stylistic features**	
Part of speech Tag: JJR	Adjective, comparative (e.g. bigger)
Part of speech Tag: NNP	Proper noun, singular (e.g. Harrison)
Structural Feature: avg_number_of_stopwords_per_sentence	Average number of stop-words per sentence
Structural feature: title_ratio_uppercase	Ratio of uppercase in title
Structural feature: title_avg_number_of_all_caps_per_sentence	Average number of all caps per sentence in title
Structural feature: title_total_number_of_sentences	Total number of sentences in title
Structural feature: title_total_number_of_characters	Total number of characters in title
Structural feature: title_total_number_of_begin_upper	Total number of words which begin with upper in title
**Psychological features**	
AFINN sentiment score^23^	A number in $[-5, 5]$, indicating the negative or positive sentiment
Laver Garry’s dictionary: CULTURE_POPULAR	Related with popular culture (e.g. media)
Loughran-McDonald’s^56^ WEAK_MODAL	Classifies the words into sentiment categories
Laver Garry’s dictionary: ECONOMY	Related with economy (accounting, earn, loan)
Laver Garry’s dictionary: INSTITUTIONS_ NEUTRAL	Related with neutral institutions (chair, scheme, voting)
RID secondary feeling: SOCIAL_ BEHAVIOR	Related with social behavior (ask, tell, call)
RID secondary feeling: TEMPORAL_ REPERE	Related with temporal references (e.g. when, now, then)
LIWC: AUXVERB^57^	Linguistic dimensions-auxiliary verbs (e.g. is, was, be, have)
LIWC: PPRON^57^	Linguistic dimensions-personal pronouns (e.g. i, you, my, me)
LIWC: THEY^57^	Linguistic dimensions-3rd pers plural (e.g. they, their, they’d)
LIWC: WE^57^	Linguistic dimensions-1st pers plural (e.g. we, us, our)
LIWC: IPRON^57^	Linguistic dimensions-impersonal pronouns (e.g. it, it’s, those)
LIWC: NEGATE^57^	Linguistic dimensions-negate (e.g. no, not, never)
LIWC: WORK^57^	Personal concerns-work (e.g. job, majors)
LIWC: RELIG^57^	Personal concerns-religion (e.g. altar, church)
LIWC: DEATH^57^	Personal concerns-death (e.g. bury, coffin, kill)
LIWC: CERTAIN^57^	Psychological processes-certainty (e.g. always, never)
LIWC: NEGEMO^57^	Psychological processes-negative emotion (e.g. hurt, ugly, nasty)
LIWC: TIME^57^	Time orientations-time (e.g. end, until, season)
LIWC: RELATIV^57^	Time orientations-relativity (e.g. area, bend, exit)
LIWC: HEAR^57^	Perceptual processes-hearing (e.g. listen, hearing)
LIWC: ACHIEV^57^	Drives and needs-achievement (e.g. win, success, better)

Continuing now with the PCA-based dimensionality reduction method with 10 Principal components, the 14 day change-point periods were detected. Specifically, four change-point locations were detected for the periods of 2011-01-19 until 2011-02-01, 2012-06-06 until 2012-06-19, 2012-10-10 until 2012-10-23 and finally for the period of 2016-06-15 until 2016-06-28. Lastly, it should be highlighted that since PCA performs dimensionality reduction and not feature selection, the resultant components cannot be directly interpreted like the lasso-based approach.

We conclude that abrupt changes in the trend of the signal are present. Four change-points have been detected when either the Lasso or the PCA based dimensionality reduction method has been employed; the estimated change-point locations for the two dimensionality reduction methods are similar. This indicates the robustness of the proposed framework in this paper, as described in the "Dimensionality reduction" and "Change-point detection" sections. It is crucial to mention that even though the detected locations are close to each other for the two methods, there are some discrepancies which occurred, mainly due to the fact that the two reduction methods obviously produce different feature sets. Furthermore, since the Lasso based reduction method provides some interpretation regarding the characteristics which the changes were perceptible the most, it was perceived that most of the detected periods had abrupt changes in at least one stylistic feature which was related to the title of the article. Finally, we observed that our dataset included numerous articles related to major political events that took place near the dates of the detected change-points (e.g. phenomena regarding the beginning of the Syrian Civil War which took place in March 2011, as well as political scandals which occurred in early October 2016, that played an important role in the 58th United States presidential election). These findings merit additional analysis and discussion in potential future works with the utilization of the DECLARE framework.

The robustness of our results has also been investigated under the scenario where the data are slightly altered. To be more precise, 5% of the observations in every time period have been randomly taken out before we carried out change-point detection; this data removal process has been repeated a total of 100 times, meaning that 100 different data sets were created, which contained approximately 95% of the values of the initial data set. The change-point detection results related to these 100 shrunken data sets are in fact very good. More specifically, 84% of the times we get either the same number of estimated change-points as in the original data set or we are at the smallest possible distance $(\pm 1)$, meaning that we estimate either 3 or 5 change-points. Furthermore, the estimated change-point locations are in a close neighbourhood of the locations obtained when the original data set was employed in our analysis.

## Conclusion

This work was initially focused on the establishment of LinCFNA, a large open dataset which consists of 534 linguistic features of time-stamped fake and real news-articles. Due to the massive amount of available articles in LinCFNA, it allows for the re-use and discovery by the research community, in order to further build upon and advance the knowledge and tools around disinformation and fake-news detection.

Furthermore, the paper proposes a framework which includes the data collection, topic classification, dimensionality reduction and change point detection procedures. The proposed framework permits the usage of specific parameters for selecting desired articles and features of a certain timeline and topic for further analysis. Ultimately, experiments and results have been carried out by employing our methods on political news articles. It has been demonstrated that most of the linearly informative characteristics of political articles are related to psychology, while interestingly, most of the significant stylistic attributes are structural features and are linked to the title of the articles. Moreover, the results of the application of change-point detection methods not only support the fact that linguistic characteristics of the political articles are evolving, but have also indicated that in several time-points, even the most informative features of the articles undergo significant changes, making the fake and non-fake articles harder to distinguish. Lastly, we observed that significant changes in the linguistic behaviour of political articles occurred on dates where major real-life political phenomena took place. In potential future work using the DECLARE framework, these findings deserve more examination and discussion.

It should be highlighted that the crawling process for extracting information from old websites or domains that changed ownership is challenging. This is due to the fact that WayBackMachine removes information of websites when a well-reasoned request has been made by the content creators or when a new ownership for the page has commenced. Another important challenge is the modern and advanced generative based models which can be used for article writing^58^. Even though that family of models can generate false content, it may be difficult to identify the validity of the content based on the writing style and linguistic features, since those models try to mimic human-like writing behaviour. This matter deserves more investigation, and in particular, with the application of the proposed framework, the evolution of linguistic features that can be extracted from the content of generation models could be studied.

Overall, the analytical and collective methods developed in this work have proved to be sensitive and beneficial for the analysis of fake news and articles in general. Through this study, we would like to encourage researchers to consider the usage of certain linguistic characteristics, as well as the linguistic alternations of fake news due to the temporal component, in order to further improve their methodologies and models in engaging fake news.

## Supplementary Information

Supplementary Information.

## Data Availability

The LinCFNA dataset along with the original text of the articles collected and the political articles’ features that lasso had produced, have all been made publicly available at the following repository: https://github.com/nikopetr/LinCFNA.
