# Supplementary material for: A Multiple change-point detection framework on linguistic characteristics of real versus fake news articles
Source: Sci Rep. 2023 Apr 13;13:6086. doi: 10.1038/s41598-023-32952-3 (PMC10100634; doi:10.1038/s41598-023-32952-3)
Supplement: Supplementary file 1 — Supplementary Information. [file 41598_2023_32952_MOESM1_ESM.pdf]

A Multiple change-point detection framework on linguistic characteristics of real versus fake news articles - Supplementary Information

Nikolas Petrou*[1], Chrysovalantis Christodoulou[1], Andreas Anastasiou†[2], George Pallis‡[1], and Marios D. Dikaiakos[1]

[1]Computer Science Department, University of Cyprus, Cyprus
[2]Department of Mathematics and Statistics, University of Cyprus, Cyprus

Supplementary Methods

In the Results Section of the main paper, we have provided an explanation of the change-point detection outcome when the first 10 principal components were used for the dimensionality reduction part of the DECLARE framework. Here, we present the results when the first 20 or 30 principal components were, instead, employed. For the former case, exactly the same, as when the method was employed on the first 10 principal components, 14-day change-point periods were detected. For the latter case, where we employed the 30 first principal components, two more 14-day change-point periods were detected, these being the periods from 2013-10-23 until 2013-11-05, and from 2014-04-23 until 2014-05-06. The estimation of two additional change-points is related to the existence of spikes in the 10 additional data sequences; such spikes can lead to the detection of possibly spurious change-points. Based on the results obtained when the first 10, 20, and 30 principal components were employed for the analysis, we conclude that our method is robust in extracting with accuracy the locations of the important changes in the given multivariate data sequence.

Supplementary Figures

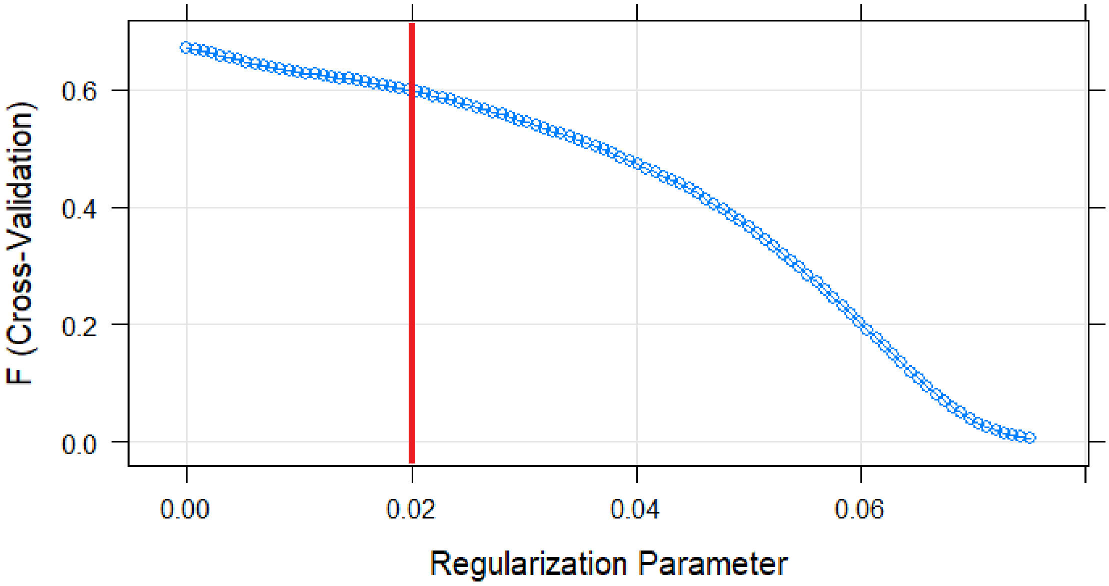

Figure S1. F1-Scores based on the 10-fold CV for the different regularization values exploited. Red line indicates the selected regularization value, which is used to select the 34 unique features

*nick.petrou.lim@gmail.com
†anastasiou.andreas@ucy.ac.cy
‡pallis.george@ucy.ac.cy

Supplementary Tables

FEATURE	SAMPLE VARIANCE
title-total-number-of-lines	0
title-psy-lg-culture-sport	0
title-psy-lg-environment	0
title-psy-lg-groups	0
title-psy-lg-law-and-order	0
title-psy-lg-values	0
title-psy-rid-primary	0
title-psy-rid-secondary	0
title-psy-rid-emotions	0
title-positions-2	0
title-hedges-hyland2005	0
text-no	0
title-no	0
title-bias-lexicon	0
psy-lg-environment	0
psy-lg-groups	0
psy-lg-law-and-order	0
psy-lg-values	0
psy-rid-primary	0
psy-rid-secondary	0
psy-rid-emotions	0
positions-2	0
text-po	0
title-po	0
bias-lexicon	0
title-factives-hooper1975	3.445583e-05
title-pos-LS	6.890928e-05
title-report-verbs	8.039324e-05
sf-ratio-digit	8.994078e-05

Table S1. Removed features with very low sample variance
